# Intellectual disability in the children of the Avon Longitudinal Study of Parents and Children (ALSPAC)

**DOI:** 10.12688/wellcomeopenres.17803.2

**Published:** 2023-01-11

**Authors:** Paul Madley-Dowd, Richard Thomas, Andy Boyd, Stanley Zammit, Jon Heron, Dheeraj Rai

**Affiliations:** 1 Centre for Academic Mental Health, Bristol Medical School, University of Bristol, Bristol, BS8 2BN, UK; 2 Population Health Sciences, Bristol Medical School, University of Bristol, Bristol, BS8 2BN, UK; 3 NIHR Biomedical Research Centre, University of Bristol, Bristol, BS8 2BN, UK; 4 MRC Centre for Neuropsychiatric Genetics and Genomics, Cardiff University, Cardiff, CF24 4HQ, UK; 5 Avon and Wiltshire Partnership NHS Mental Health Trust, University of Bristol, Bristol, BA1 3QE, UK

**Keywords:** Intellectual disability, Neurodevelopment, ALSPAC

## Abstract

**Background:** Intellectual disability (ID) describes a neurodevelopmental condition involving impaired cognitive and functional ability. Here, we describe a multisource variable of ID using data from the Avon Longitudinal Study of Parents and Children (ALSPAC).

**Methods: **The multisource indicator variable for ID was derived from i) IQ scores less than 70 measured at age 8 and at age 15, ii) free text fields from parent reported questionnaires, iii) school reported provision of educational services for individuals with a statement of special educational needs for cognitive impairments, iv) from relevant READ codes contained in GP records, iv) international classification of disease diagnoses contained in electronic hospital records and hospital episode statistics and v) recorded interactions with mental health services for ID contained within the mental health services data set. A case of ID was identified if two or more sources indicated ID. A second indicator, labelled as “probable ID”, was created by relaxing the cut off in IQ scores to be less than 85. An indicator variable for known causes of ID was also created to aid in aetiological studies where ID with a known cause may need to be excluded.

**Results:** 158 of 14,370 participants (1.10%) were indicated as having ID by two or more sources and 449 (3.12%) were indicated as having probable ID when the criteria for IQ scores was relaxed to less than 85. There were 476 participants (3.31%) with 1 or fewer sources of available information on ID; these participants had their multisource variable set to missing. The number of cases of ID with known cause was 31 (0.22% of the cohort, 19.6% of those with ID).

**Conclusions**: The multisource variable of ID can be used in future analyses on ID in ALSPAC children.

## Introduction

Intellectual disability (ID) is a developmental condition defined as having an arrested or incomplete development of the mind alongside functional impairment in facets that contribute to overall intelligence such as cognition, language and social ability ^1^. ID manifests during the developmental period and is not the result of later changes to the brain as a result of injury or disease.

There are several challenges in defining ID in practice, particularly in relation to the language used. Several terms are used in the UK including learning disability, learning difficulties, developmental disorder (or delay) and special educational needs ^2^. Confusion can arise as these phrases are components of other, separate concepts. For example, specific learning disability refers to dyslexia or dyscalculia, while learning difficulty can refer to intellectual disability or a specific learning disability. It is important to note that those with ID may also have a specific learning disability. Further challenges arise in the definitions used between studies based in different global regions. In the USA the phrase “intellectual disability” carries the same meaning as “learning disability” in the UK, while use of the phrase “learning disability” in the USA refers to what would be described as a “specific learning disability” in the UK.

In a healthcare setting, several diagnostic criteria including the International Classification of Diseases, Version 10 (ICD-10) ^1^ and Diagnostic and Statistical Manual of Mental Disorders, 4 ^th^ edition (DSM-IV) ^3^ define ID using an intelligence quotient (IQ) score of less than 70; equivalent to 2 standard deviations (SD) less than the assumed population average of 100, alongside functional impairments. The Diagnostic and Statistical Manual of Mental Disorders, 5 ^th^ Edition (DSM-5) ^4^, states that IQ tests will generally be measured with an error of around 5 points and therefore scores between 65 and 75 may indicate ID. The definition used will greatly affect the prevalence of ID in studies. For example, Cooper *et al.* ^5^ note that the proportion of the population expected to lie in the range of IQ scores between 70 and 75 (2.5%), is greater than the proportion of the population expected to have ID using scores less than 70 as a cut off (2.28%). The educational system in the UK uses an even less stringent cut off, IQ less than 85 (equivalent to 1 standard deviation lower than the population average), to indicate “mild learning difficulty” ^6, 7^.

It has been argued that ID should not be defined on the basis of IQ test scores alone ^7, 8^ due to the instability of the measure on the basis of mood and fatigue, potential to be influenced by learning or rehearsal, and tests that are largely centred around Western cultural understanding that may have important implications, particularly for migrants. The ICD-10 and DSM-5 also use social functioning and age of onset for diagnosis. Those who have an IQ less than 70 but are able to function without assistance by this definition are not considered to have ID in relation to clinical services. Cooper *et al.* ^5^ provide examples such as living independently and holding a job as meeting this criteria of functioning without assistance. Such a definition means that ID is not necessarily stable throughout the life. Those with ID do learn throughout the lifetime, and some of those who require significant support during school age years may go on to learn to live independently. 

Intellectual disability has been under-researched in large epidemiological investigations leading to a relative lack of understanding of both its aetiology and consequences. The Avon Longitudinal Study of Parents and Children (ALSPAC) has recorded data in the form of questionnaires, biological samples, and genetic information for several thousand participants from gestation in the early 1990s to the present day. The cohort therefore provides an opportunity to explore both early life causes of ID and its long-term outcomes.

Our goal was to derive a multi-sourced measure of ID for participants of ALSPAC. Data is available from IQ tests measured by trained study fieldworkers at different ages during participant visits to the ‘study assessment clinic’. However, participation in ALSPAC, and at these at clinics, may be influenced by having ID. This pattern of missing data is likely to lead to biases in complete case analyses ^9, 10^ and in analyses that attempt to address missing data such as multiple imputation ^11, 12^. Data linkage to school reported statements of special educational needs and health service reported data on diagnoses and interactions with mental health services can be used to supplement the missing information. In this Data Note, we describe the processes used to derive indicator variables of ID which can be used by researchers in their own studies.

## Methods

### ALSPAC sample

The ALSPAC cohort ^13, 14^ recruited 14,541 pregnant women resident in Avon, UK with expected dates of delivery 1st April 1991 to 31st December 1992. Each enrolled mother either returned at least one questionnaire or attended a “Children in Focus” clinic by 19/07/99. The core sample of pregnancies (also referred to as Phase I) contained a total of 14,676 fetuses that resulted in 14,062 live births; 13,988 of these index children were alive at 1 year of age.

Attempts were made to bolster the initial core sample with eligible cases who had failed to join the study originally. These attempts were made in 1999 when the oldest children were approximately 7 years of age (Phase II recruitment; 456 children recruited), opportunistically from 1999–2012 (Phase III; 262 children recruited) and then from 2012 onwards with specific focus on recruiting second generation pregnancies (Phase IV; 195 index children recruited) ^15^. The phases of enrolment are described in more detail in the cohort profile paper and its update ^13, 14^.

Data has been collected on the cohort since its inception and is still ongoing. The mothers, their partners and the index child have been followed up using clinics, questionnaires, and links to routine data. The study website contains details of all the data that is available through a fully searchable data dictionary. From age 18, study children were sent 'fair processing' materials describing ALSPAC’s intended use of their health and administrative records and were given clear means to object via a written form. This was an ‘opt out’ approach, meaning linkage was attempted for all participants, except those who objected and those who were not sent fair processing materials. Where ‘opt in’ consent became practicable (e.g., when a participant attended a study assessment visit) then this was collected by a trained fieldworker.

**Figure 1.**
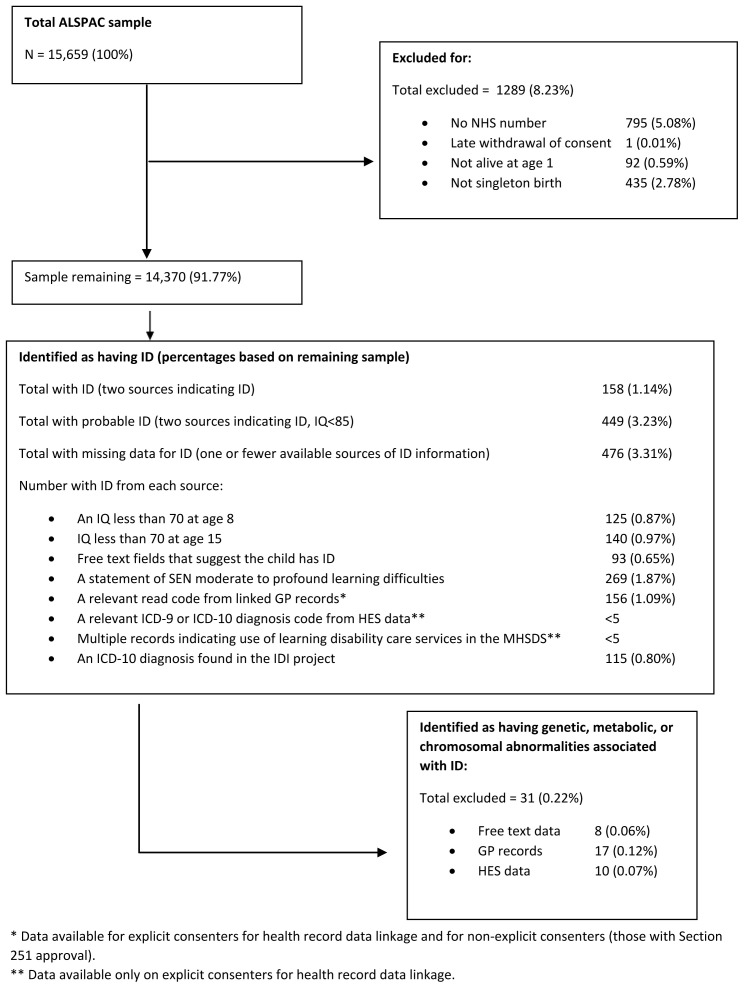
Flowchart of cohort derivation.

There were 15,659 total ALSPAC mother-child pairs across Phase I-IV recruitment. Of these, 795 had no NHS number and so could not be linked to the UK Secure eResearch Platform (UKSeRP) where the data were held, 1 participant withdrew consent at this stage. Of the remaining 14,863 participants, 92 were not alive at 1 year of age and 435 were not singleton births (not mutually exclusive groups). On removal of these, a sample of 14,370 mother-child pairs remained. A cohort flow diagram is presented in Figure 1, that describes the exclusion process for each stage of the study.

### Data sources for ID indicator

Data from ALSPAC sources included measures of IQ taken at age 8 and 15 and free text fields in child-based questionnaires where the responder could record additional information. The linked sources included the Pupil Level Annual School Census (PLASC) which recorded the provision of educational services for individuals with statements of special educational needs (SEN), General Practitioner (GP) records which recorded Read codes related to ID, Hospital Episode Statistics (HES) data which recorded International Classification of Disease (ICD) ^1^ diagnosis codes for ID and the Mental Health Services Data Set (MHSDS) which contains information on interactions with mental health services for reasons related to ID. Data linkage has previously been undertaken in the Identification of Developmental Impairments (IDI) project led by Emond ^16^ which identified neurodevelopmental disorders up to a maximum age of 11 years using ICD-10 diagnoses and statements of SEN. Further details of each source of information is provided in the subsections below.

Availability of linked health records (GP records, HES data and MHSDS data) was divided into four groups: (i) those who had explicitly consented to data linkage (5,063 individuals; 35.23%), (ii) those who had not explicitly consented to data linkage (7,358 individuals; 51.20%), (iii) those who had explicitly refused consent for data linkage (359 individuals; 2.50%), and (iv) those who had no data linkage available (1,590 individuals; 11.06%). A Confidentiality Advisory Group (CAG) application ^17^ was made to obtain access to the information of those who had not explicitly consented to data linkage (group ii) via use of Section 251 of the National Health Service Act 2006 ^18^. The CAG application, submitted by the ALSPAC data linkage team ^19^, via the Integrated Research Application System ^20^ (CAG reference: 20.CAG/0056; IRAS project ID: 268410) and aligned NHS Data Sharing Agreements, support the use of GP records for this study but not HES or MHSDS data. As a result, data are available on all linked health records for explicit consenters (group i), and on GP records only for non-explicit consenters (group ii).

***IQ scores.*** IQ at age 8 years was measured using a short form of the Wechsler Intelligence Scale for Children - III ^21^ which consisted of alternate items for all subtests except the coding subtest (which was administered in full) as part of a half day battery of mainly psychological and psychometric testing. IQ at age 15 years was measured using the Wechsler Abbreviated Scale of Intelligence ^22^ as part of a 4 hour battery of testing. Data was available for 7,113 (49.50% of total ALSPAC sample after exclusions) individuals at age 8 and for 5,116 (35.60%) individuals at age 15. From the IQ scores binary variables were created indicating if IQ was below 70 at each age. A second variable was created indicating a less stringent cut off of IQ below 85, equivalent to one population standard deviation below the assumed population average of 100.

***Free text fields.*** ALSPAC contains free text responses to many questions answered by participants and their guardians across the lifetime of the study. For example, at age 9 guardians (typically mothers) of participant children were asked whether the children had been identified as having any particular problems at school and to describe in text each type of school problem. A search was performed across all free text fields contained in ALSPAC for terms related to ID (see Table 1 for the search terms used and number of hits). A review of all free text responses for each individual identified with relevant free text fields (n=203) was performed to check if the text indicated whether the child was likely to have ID or not. Any queries were checked by a clinician who specialises in neurodevelopmental disorders (author DR). We did not classify individuals as having ID if the search terms identified specific learning difficulties (e.g., dyslexia or difficulties specific to maths and literacy ability) or where the terms identified individuals as explicitly not having a learning disability. Following the review of all free text fields for each identified individual, 94 individuals were classed as having ID and 109 individuals were classed as not having ID. Free text data was available for 12,722 individuals in the sample. 

**Table 1.** Search terms and number of hits for free text fields.

Search term	Number of Hits in ALSPAC
intellectual disability	0
developmental disabilities	0
intellectual disab	0
developmental disab	0
learning disab	15
mental retard	<5
mental handicap	0
handicap	5
intellectual	5
retard	7
learning disability	6
learning disabled	<5
learning difficulties	137
learning difficulty	41
difficulty learning	<5
mental disability	0
mentally disabled	<5
mentally retarded	0
mental retardation	<5
low IQ	<5
development delay	20
developmental delay	29

***Pupil level annual school census (PLASC) records of provision for special educational needs.*** Educational provision for children with SEN statements falling under the category “cognition and learning needs” ^23^ were used to indicate ID. Records of these provisions were made in 2003/4 when the vast majority of the sample children were in school years 6–8 (ages 11–13). We identified all individuals within the category who had a statement for moderate to profound learning difficulties as being a case of ID. The cognition and learning needs category also includes individuals with specific learning difficulties related to problems learning to read, write, spell or manipulate numbers. This latter group were not included as having ID unless they also had a statement for moderate to profound learning difficulties.

PLASC data were available for 10,349 (72.02%) of the sample. Those who did not have a PLASC record either did not attend state school in England (includes those attending independent schools, schools outside of England or those educated at home) or could not be matched (for example if their name was changed without ALSPAC being informed) or were not included in the linkage sample as no legal basis could be established. Absence of PLASC information may therefore be associated with ID status and/or enrolment in state provided education.

***GP records.*** GP records contain coded information in the form of Read codes ^24, 25^. These are a hierarchically coded thesaurus of clinical terms that have been in use by the NHS since 1985. The codes are entered into a computerised system by clinicians or practice staff from general practice or secondary care consultations. A list of version 2 Read codes was created by checking for terms related to intellectual disability or its synonyms using the UK Read Browser, previously accessible from NHS digital’s Technology Reference data Update Distribution. The list of Read codes identified was cross checked against a list of codes selected in a previous study looking at incidence of mental illness and challenging behaviour in individuals with ID ^26^. Terms that appeared in either list were used (see Table 2 for the Read codes used). Data was available for 12,421 individuals (86.44%) of the sample. Those who did not have a GP record either received primary care outside of England or Wales or via a private (non-NHS) provider; individuals whose GP did not approve the studies extraction of their record; or could not be matched (due to linkage failure); or were not included in the linkage sample as no legal basis could be established (those who objected or where no fair processing could occur). Absence of GP records may therefore be associated with ID status.

**Table 2.** Read codes used to indicate intellectual disability in GP records.

Read code	Description
13Z4E	Learning difficulties
6664	Mental handicap problem
69DB	Learning disability health examination
8Ce6	Preferred place of care - learning disability unit
8H4f	Referral to learning disabilities psychiatrist
8Hg2	Discharge from learning disability team
8HHP	Referral to learning disability team
918e	On learning disability register
94Z9	Preferred place of death: learning disability unit
9HB	Learning disabilities administration status
9HB0	Learning disabilities health action plan declined
9HB1	Learning disabilities health action plan offered
9HB2	Learning disabilities health action plan reviewed
9HB3	Learning disabilities health assessment
9HB4	Learning disabilities health action plan completed
9HB5	Learning disabilities annual health assessment
9HB6	Learning disabilities annual health assessment declined
9HB7	Did not attend learning disabilities annual health check
9hL	Exception reporting: learning disability quality indicators
9hL0	Excepted from learning disability quality indicators: informed dissent
9hL1	Excepted from learning disability quality indicators: patient unsuitable
9mA	Learning disability annual health check invitation
9mA0	Learning disability annual health check verbal invitation
9mA1	Learning disability annual health check telephone invitation
9mA2	Learning disability annual health check letter invitation
9mA20	Learning disability annual health check invtation 1st letter
9mA21	Learning disability annual health check invtation 2nd letter
9mA22	Learning disability annual health check invtation 3rd letter
9N0y	Seen in learning disabilities clinic
CLEVALE7	Learning difficulties annual check done
E3	Mental retardation
E30	Mild mental retardation, IQ in range 50–70
E30-1	educationally subnormal
E31	Other specified mental retardation
E310	Moderate mental retardation, IQ in range 35–49
E311	Severe mental retardation, IQ in range 20–34
E312	Profound mental retardation with IQ less than 20
E31z	Other specified mental retardation NOS
E3y	Other specified mental retardation
E3z	Mental retardation NOS
EMISNQLE18	Learning disability monitoring in primary care
EMISNQLE5	Learning disability
EMISQRE10	Referral to learning disability team
Eu7	[X]Mental retardation
Eu70	[X]Mild mental retardation
Eu700	[X]Mld mental retard with statement no or min impairm behav
Eu701	[X]Mld mental retard sig impairment behav req attent/treatmt
Eu70y	[X]Mild mental retardation, other impairments of behaviour
Eu70z	[X]Mild mental retardation without mention impairment behav
Eu71	[X]Moderate mental retardation
Eu710	[X]Mod mental retard with statement no or min impairm behav
Eu711	[X]Mod mental retard sig impairment behav req attent/treatmt
Eu71y	[X]Mod retard oth behav impair
Eu71z	[X]Mod mental retardation without mention impairment behav
Eu72	[X]Severe mental retardation
Eu720	[X]Sev mental retard with statement no or min impairm behav
Eu721	[X]Sev mental retard sig impairment behav req attent/treatmt
Eu72-1	[x]Severe mental subnormality
Eu72y	[X]Severe mental retardation, other impairments of behaviour
Eu72z	[X]Sev mental retardation without mention impairment behav
Eu73	[X]Profound mental retardation
Eu730	[X]Profound mental retardation with the statement of no, or minimal, impairment of behaviour
Eu731	[X]Profound ment retard sig impairmnt behav req attent/treat
Eu73y	[X]Profound mental retardation, other impairments of behavr
Eu73z	[X]Prfnd mental retardation without mention impairment behav
Eu7y	[X]Other mental retardation
Eu7y0	[X]Oth mental retard with statement no or min impairm behav
Eu7y1	[X]Oth mental retard sig impairment behav req attent/treatmt
Eu7yy	[X]Other mental retardation, other impairments of behaviour
Eu7yz	[X]Other mental retardation without mention impairment behav
Eu7z	[X]Unspecified mental retardation
Eu7z0	[X]Unsp mental retard with statement no or min impairm behav
Eu7z1	[X]Unsp mentl retard sig impairment behav req attent/treatmt
Eu7zy	[X]Unspecified mental retardatn, other impairments of behav
Eu7zz	[X]Unsp mental retardation without mention impairment behav
Eu814	[X]Moderate learning disability
Eu815	[X]Severe learning disability
Eu816	[X]Mild learning disability
Eu817	[X]Profound learning disability
Eu81z	[X]Learning disorder NOS
Eu81z-1	[x]learning disability nos
Eu81z-2	[X]Learning disorder NOS
Eu841	[X]Mental retardation with autistic features
Eu844	[X]Overactive disorder assoc mental retard/stereotype movts
FUNDHME1	Mental handicap psyc referral
HNG0150	[rfc] learning disabilities
HNG0625	[rfc] learning disability
PKyG	Mental retardation, congenital heart disease, blepharophimosis, blepharoptosis and hypoplastic teeth
R034E	[D]Developmental delay
R034y	[D]Global retardation
Z7CBE	Intellectual functioning disability
Z7CD2	Learning difficulties
ZL1B5	Under care of psychiatrist for mental handicap
ZL5B5	Referral to psychiatrist for mental handicap
ZL9D5	Seen by psychiatrist for mental handicap
ZLD2f	Discharge by psychiatrist for mental handicap
ZLE94	Discharge from mental handicap psychiatry service
ZS34	Learning disability
ZV623	[V]Educational handicap

***Hospital episode statistics.*** Details of all admissions, attendances at accident and emergency and outpatient appointments at NHS hospitals in England are collected in the centralised, national, HES database ^27^. Data for admitted patients are available from April 1997, for outpatient appointments from April 2003 and for accident and emergency attendances from April 2007. This means that data from these sources are available from when the participants were 5–6, 11–12 and 15–16 years of age respectively. The HES dataset recorded all diagnoses up until 1995 using ICD-9 and all diagnoses in subsequent years as ICD-10 codes ^28^. Diagnoses of 317-319 (ICD-9) and F70-F79 (ICD-10) made during hospital interactions were used to indicate ID. Data was available for the 5,063 individuals (35.21% of the sample) who had explicitly consented to data linkage of health records and who had presented for hospital care in England. All obtained diagnoses of ID were found in admitted patient records and none were found in either of the outpatient of accident and emergency records.

***Mental health services data set.*** The MHSDS collects data on all interactions between patients and specialist secondary mental health care services ^29^. Patients are assigned to mental health clusters using the Health of the Nation Outcome Scales ^30^ which can be used to indicate the nature of the mental health care. Information regarding intellectual disability can be found within care clusters 18-21 which relate to cognitive impairment. All individuals that had more than one recorded final clinician allocated cluster related to cognitive impairment were indicated as having ID. Less than 5 cases were indicated using this method. All were contained within cluster 18.

MHSDS data was only available for 188 individuals (1.31% of the total sample) who had a relevant Read code found in GP records or ICD code found in HES data. The sample for who MHSDS data was available was therefore a subsample of the explicitly consenting sample of 5,063 individuals who received community mental health care in England.

***IDI project.*** The IDI project has been described in detail elsewhere ^16^. Briefly, the project identified individuals in the ALSPAC cohort with any form of developmental delay as defined by ICD-10 classification. Information on diagnoses was obtained from computerised medical records of NHS trusts in the local Bristol area between 1991 and 2003 (North Bristol Trust, United Bristol Healthcare Trust, Weston Area Health Trust and Royal United Hospital, Bath) and from the Child Health computer system (shared across all NHS trusts in Bristol) for all children identified as having special educational needs between 1993 and 2003. This SEN classification was obtained through linkage to local authority held education records. A team of three researchers searched the hospital medical records (inpatient and outpatient) and the community child-health records to identify relevant diagnoses made after multidisciplinary assessment. For the current project, diagnoses codes of F70-F79 were used to select those with a diagnosis of ID.

It was not possible to determine the exact overlap between the IDI project sample and the analysis sample of the current project. This was due to the data retained from the IDI project only containing information on those who had an identified diagnosis and not all those for whom medical records were available at the time of the project. The documentation for the IDI project (which can be obtained from the ALSPAC ‘useful data’ repository) states that 13,898 of the 14,062 live born individuals who make up the ALSPAC Phase 1 sample were eligible for the IDI project: this larger sample size reflects less stringent governance requirements of the time. It was therefore assumed that data was available on IDI diagnoses for all Phase 1 ALSPAC participants. 

### Multi-sourced indicator of ID

The information available to create a multi-sourced indicator of ID were therefore the following eight items:

1. An IQ less than 70 at age 8
2. IQ less than 70 at age 15
3. Free text fields that suggest the child has ID
4. A statement of SEN for moderate to profound learning difficulties
5. A relevant Read code from linked GP records
6. A relevant ICD-9 or ICD-10 diagnosis code from HES data
7. Multiple records indicating use of learning disability care services in the MHSDS
8. An ICD-10 diagnosis found in the IDI project

A case of ID was identified if two or more of the eight criteria were met. We defined a second variable, labelled as “probable ID” using the same criteria as above except that the threshold for ID from the IQ scores was relaxed to 85 (1 SD lower than the population average). This was done to be closer aligned with the definition of borderline ID used by the UK educational system. Where a participant had observed data in only one or fewer sources, they were considered to have missing data for the multi-sourced indicator of ID.

### Known causes of ID – a tool for exclusion criteria

Individuals who have a genetic, metabolic, or chromosomal abnormality that is associated with ID constitute a group in which ID is likely regardless of environmental exposure. Such a group may need to be excluded in analyses investigating the aetiology of ID. Genetic, metabolic, or chromosomal abnormalities associated with ID were identified using free text information, GP records and HES data. The free text records of individuals with text relevant to ID were screened for mentions of known genetic causes of ID. Read codes and ICD codes for genetic disorders related to ID were obtained from GP records and HES data. A list of the codes used is presented in Table 3. If a participant had any of these codes, they were provided with a “known cause of ID” flag. In total 31 participants had a known cause of ID resulting from genetic, metabolic, or chromosomal abnormalities (8 using free text data, 17 using GP records and 10 using HES data).

**Table 3.** Codes (Read and ICD) used to identify genetic, chromosomal and metabolic abnormalities.

Source (code type)	Code	Description
GP records (Read code)	1JB0	Suspected Downs syndrome
	677C4	Carrier of fragile X gene mutation
	C301	Phenylketonuria
	EMISNQCA42	Cause of learning disabilities: down's syndrome
	Eu842	[X]Rett's syndrome
	F1y0	Fragile X associated tremor ataxia syndrome
	PJ0	Down's syndrome - trisomy 21
	PJ00	Trisomy 21, meiotic nondisjunction
	PJ01	Trisomy 21, mosaicism
	PJ02	Partial trisomy 21 in Down's syndrome
	PJ0-2	trisomy 21
	PJ0-98	Down's syndrome
	PJ0z	Down's syndrome NOS
	PJ1..	Patau's syndrome - trisomy 13
	PJ10.	Trisomy 13, meiotic nondisjunction
	PJ11.	Trisomy 13, mosaicism
	PJ12.	Trisomy 13, translocation
	PJ1z.	Patau's syndrome NOS
	PJ2	Edward's syndrome - trisomy 18
	PJ20	Trisomy 18, meiotic nondisjunction
	PJ21	Trisomy 18, mosaicism
	PJ22	Partial trisomy 18 in Edward's syndrome
	PJ2z	Edward's syndrome NOS
	PJ30.	Deletion of long arm of chromosome 21
	PJ31	Cri-du-chat syndrome
	PJ32.	Deletion of short arm of chromosome 4
	PJ330	Deletion of long arm of chromosome 13
	PJ331	Deletion of long arm of chromosome 18
	PJ332	Deletion of short arm of chromosome 18
	PJ333	Smith-Magenis syndrome
	PJ334	Jacobsen syndrome
	PJ36.	Whole chromosome monosomy, meiotic nondisjunction
	PJ37.	Whole chromosome monosomy, mosaicism
	PJ3y0	Shprintzen syndrome
	PJ50.	Whole chromosome trisomy syndromes
	PJ500	Trisomy 6
	PJ501	Trisomy 7
	PJ502	Trisomy 8
	PJ503	Trisomy 9
	PJ504	Trisomy 10
	PJ505	Trisomy 11
	PJ506	Trisomy 12
	PJ507	Other trisomy C syndromes
	PJ508	Trisomy 22
	PJ50w	Whole chromosome trisomy, meitotic nondisjunction
	PJ50x	Whole chromosome trisomy, mitotic nondisjunction
	PJ50y	Other specified whole chromosome trisomy syndrome
	PJ50z	Whole chromosome trisomy syndrome NOS
	PJ510	Major partial trisomy
	PJ523	Triploidy
	PJ524	Polyploidy
	PJ534	Individual with autosomal fragile site
	PJ5y.	Pseudotrisomy 18
	PJ636	Turner's phenotype, ring chromosome karyotype
	PJ71.	Klinefelter's syndrome, male with more than two X chromosomes
	PJ73.	Klinefelter's syndrome, XXYY
	PJ9..	Mowat-Wilson syndrome
	PJyy2	Fragile X chromosome
	PJyy4	Fragile X syndrome
	PK5	Tuberous sclerosis
	PK61	Sturge-Weber syndrome
	PKy0	Prader-Willi syndrome
	PKy0-1	Prader-Willi syndrome
	PKy0-2	Prader-Willi syndrome
	PKy03	Weaver syndrome = Sotos syndrome
	PKy4	William syndrome
	PKy60	Cornelia de Lange syndrome
	PKy80	Noonan's syndrome
	PKy92	Menke's syndrome
	PKy93	Prader - Willi syndrome
	PKy94	Zellweger's syndrome
	PKy95	Biemond's syndrome
	PKyz.	Cockayne's syndrome
	PKyz0	Ullrich - Feichtiger syndrome, chimaera
	PKyz5	Angelman syndrome
	PKyz7	Angelman syndrome
	ZC2C6	Dietary advice for phenylketonuria
HES data (ICD-9 codes)	270.0	Disturbances of amino-acid transport
	270.1	Phenylketonuria [PKU]
	270.2	Other disturbances of aromatic amino-acid metabolism
	270.3	Disturbances of branched-chain amino-acid metabolism
	270.4	Disturbances of sulphur-bearing amino-acid metabolism
	270.5	Disturbances of histidine metabolism
	270.6	Disorders of urea cycle metabolism
	270.7	Other disturbances of straight-chain amino-acid metabolism
	270.8	Other specified disorders of amino-acid metabolism
	270.9	Unspecified disorder of amino-acid metabolism
	271.8	Other specified disorders of carbohydrate transport and metabolism
	272.8	Other disorders of lipoid metabolism
	277.81	Primary carnitine deficiency
	277.82	Carnitine deficiency due to inborn errors of metabolism
	277.83	Iatrogenic carnitine deficiency
	277.84	Other secondary carnitine deficiency
	277.85	Disorders of fatty acid oxidation
	277.86	Peroxisomal disorders
	277.89	Other specified disorders of metabolism
	279.11	Digeorge's syndrome
	330.8	Other specified cerebral degenerations in childhood
	751.60	Unspecified anomaly of gallbladder, bile ducts, and liver
	751.69	Other anomalies of gallbladder, bile ducts, and liver
	758.0	Down's syndrome
	758.1	Patau's syndrome
	758.2	Edwards' syndrome
	758.31	Cri-du-chat syndrome
	758.32	Velo-cardio-facial syndrome
	758.33	Other microdeletions
	758.39	Other autosomal deletions
	758.4	Balanced autosomal translocation in normal individual
	758.5	Other conditions due to autosomal anomalies
	758.6	Gonadal dysgenesis
	758.7	Klinefelter's syndrome
	758.81	Other conditions due to sex chromosome anomalies
	758.9	Conditions due to anomaly of unspecified chromosome
	759.5	Tuberous sclerosis
	759.81	Prader-Willi syndrome
	759.83	Fragile X syndrome
	759.89	Other specified congenital anomalies
HES data (ICD-10 codes)	D82.1	DiGeorge syndrome
	E70	Disorders of aromatic amino-acid metabolism
	E71	Disorders of branched-chain amino-acid metabolism and fatty-acid metabolism
	E72	Other disorders of amino-acid metabolism
	F84.2	Rett's syndrome
	Q44.7	Alagille syndrome
	Q85.0	Neurofibromatosis (non-malignant)
	Q85.1	Tuberous sclerosis
	Q87.1	Prader-Willi syndrome
	Q87.2	Rubinstein-Taybi syndrome
	Q89.8	Williams syndrome
	Q90	Down syndrome
	Q91	Trisomy 18 and Trisomy 13
	Q92	Other trisomies and partial trisomies of the autosomes, not elsewhere classified
	Q93	Monosomies and deletions from the autosomes, not elsewhere classified
	Q95	Balanced rearrangements and structural markers, not elsewhere classified
	Q96	Turner's syndrome
	Q97	Other sex chromosome abnormalities, female phenotype, not elsewhere classified
	Q98	Other sex chromosome abnormalities, male phenotype, not elsewhere classified
	Q99	Other chromosomal abnormalities, not elsewhere specified

## Assessment of validity

### Individual sources of ID

**Figure 2.**
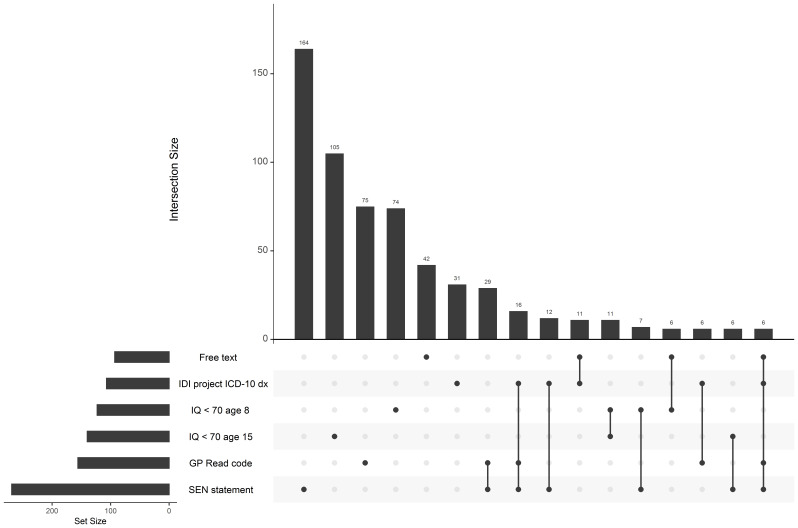
Intersection between sources indicating an intellectual disability. Intersecting groups with counts ≤5 are not shown.

Figure 2 shows the intersection of sources indicating an ID for groups with counts greater than 5 (showing unique intersections where participants are not indicated as having ID by any other source). This information is further explored in Table 4 which i) shows the number of individuals with ID indicated by each source, ii) the number of individuals with each combination of sources of ID (regardless of wider set intersections) and iii) the 95% confidence interval (CI) of the odds ratio (OR) for having ID for each source according to whether participants had another source indicating ID (given that data is available from both sources).

**Table 4.** Cross tabulation of ID obtained from each source.

ID Source	Total with data available	Total with ID indicated by source	IQ < 70 at age 8	IQ < 85 at age 8	IQ < 70 at age 15	IQ < 85 at age 15	Free text	SEN statement	GP Read code	HES ICD 9/10 diagnosis	MHSDS code	IDI project ICD-10 diagnosis
IQ < 70 at age 8	7113	125		a	15.3 - 48.4	13.5 - 67.4	18.5 - 74.4	17.8 - 53.0	12.7 - 55.7	21.5 - 5014.1	0.2 - 108.9	81.5 - 492.7
IQ < 85 at age 8	7113	858	125		14.0 - 31.8	9.0 - 14.3	10.7 - 46.5	9.1 - 24.8	7.2 - 27.4	a	a	27.4 - 572.5
IQ < 70 at age 15	5116	140	21	69		a	10.6 - 50.4	11.0 - 40.4	9.3 - 45.6	2.1 - 1380.2	a	15.0 - 122.7
IQ < 85 at age 15	5116	1207	52	287	140		5.5 - 34.5	4.6 - 18.5	1.7 - 7.5	0.2 - 102.5	a	9.2 - 828.8
Free text	12436	93	14	28	11	23		15.8 - 47.9	30.0 - 90.9	24.8 - 1844.6	0.7 - 54.6	51.4 - 141.2
SEN statement	10349	269	23	48	16	33	22		35.2 - 74.4	28.5 – 4628.6	0.2 – 54.1	91.8 – 314.2
GP Read code	12421	156	11	24	10	15	22	65		55.1 - 9045.3	0.0 - 1.7	72.2 - 196.4
HES ICD9/10 dx	5063	≤5	≤5	≤5	≤5	≤5	≤5	≤5	≤5		a	35.2 - 2724.4
MHSDS code	188	≤5	≤5	≤5	≤5	≤5	≤5	≤5	≤5	≤5		0.4 - 31.5
IDI project ICD-10 dx	13512	115	19	24	8	14	29	50	39	≤5	≤5	

Lower triangle of the table provides cross tabulation of the number of individuals with both sources indicating ID. Where counts are ≤5, the count may be equal to 0. Upper triangle of the table provides the lower and upper bounds of the 95% confidence interval for the OR obtained from the 2×2 table of the sources for ID identification. a – indicates that the confidence interval could not be calculated due to perfect prediction.

The most common combination of sources for ID were, in order, i) SEN statement and GP Read codes, ii) SEN statement, GP Read codes and an IDI project diagnosis, iii) SEN statement and IDI project diagnosis, iv) an IDI project diagnosis and free text information and v) IQ < 70 at age 8 and 15. Despite being one of the most common combinations, an IQ less than 70 was not commonly indicated by both IQ tests at age 8 and 15. Instead, it was more common to have an IQ less than 70 on one test and an IQ less than 85 on the other. An IQ less than 85 was common on both tests. Diagnosis from the IDI project seemed to be the strongest predictor of having ID indicated by other sources according to ORs. Both the HES and MHSDS sources indicated fewer than 5 cases of ID each, and therefore do not contribute much information to the multi-sourced variables.

The distribution of available IQ scores for those with ID indicated by each source of information is presented in Table 5. The mean IQ at age 8 was less than 70 among those who were indicated as having ID from the IDI project and from HES data but was greater than 70 for those with ID indicated by free text data, SEN statements and GP records. This may suggest that different severities of ID are being identified by the different sources of information. It is, however, also possible that those with lower IQs were selectively underrepresented at the collection of the IQ data, in questionnaire data and/or in the linked education records. If this is the case then the average IQ at age 8 for those with ID indicated by these sources may be lower than 70, had the missing IQ information been available. 

**Table 5.** Distribution of IQ for each source of ID.

			IQ at age 8					IQ at age 15				
Source	Description	N with ID according to source	N with IQ data	<70, N(%)	70–84, N(%)	≥85, N(%)	Mean (SD)	N with IQ data	<70, N(%)	70–84, N(%)	≥85, N(%)	Mean (SD)
Free text	Free text data in ALSPAC questionnaires indicating ID	93	38	14 (36.84)	14 (36.84)	10 (26.32)	75.26 (16.82)	29	11 (37.93)	12 (41.38)	6 (20.69)	73.31 (14.52)
SEN statement	Statement of special educational needs from the PLASC	269	72	23 (31.94)	25 (34.72)	24 (33.33)	77.57 (16.30)	44	16 (36.36)	17 (38.64)	11 (25.00)	74.95 (14.52)
GP records	A Read code related to ID found in GP records	156	38	11 (28.95)	13 (34.21)	14 (36.84)	80.45 (21.20)	29	10 (34.38)	5 (17.24)	14 (48.28)	77.55 (15.43)
HES data	An ICD-9 or ICD-10 diagnosis recorded in HES data	≤5	≤5				63.33 (16.92)					a
MHSDS data	Multiple records indicating use of learning disability care services	≤5					a					a
IDI data	ICD-10 diagnosis identified	115	26	19 (73.08)	≤5	≤5	63.58 (12.28)	15	8 (53.33)	a	a	65.13 (10.97)

Data on the right hand side of the table are for those who have an IQ measurement available. ^a^ Count too low to be presented. For columns indicating a count the value may be 0.

### Multi-sourced variables of ID

Of the sample of 14,370 individuals, 158 (1.1%) were indicated as having ID by two or more sources and 449 (3.1%) were indicated as having probable ID when the criteria for IQ scores was relaxed to less than 85. Counts of participants with each number of sources of available data and number of sources indicating ID and probable ID are displayed in Table 6. If the participant had one or fewer sources of information available (irrespective of whether the single source indicated ID) they were considered to have missing data for ID; 476 participants (3.3%) were considered to have missing data using this definition. Ten of these 476 individuals (2.1%) had one source indicating ID but no other sources of information available.

**Table 6.** Distribution of the number of available sources for ID and sources indicating ID.

X - Number of variables	Count (%) of participants with X sources available for ID information	Count (%) of participants with X sources indicating ID	Count (%) of participants with X sources indicating probable ID
0	53 (0.37)	13,711 (95.42)	12,232 (85.12)
1	423 (2.94)	501 (3.49)	1,689 (11.75)
2 (minimum number to be able to indicate ID)	1,250 (8.70)	102 (0.71)	371 (2.58)
3	1,929 (13.42)	33 (0.23)	52 (0.36)
4	3,431 (23.88)	16 (0.11)	15 (0.10)
5	2,447 (17.03)	≤5	8 (0.06)
6	2,357 (16.40)	≤5	≤5
7	2,453 (17.07)	≤5	≤5
8	27 (0.19)	≤5	≤5

Where counts are ≤5, the count may be equal to 0.

Individuals with ID and probable ID indicated by the multi-sourced variables were compared to those not indicated as having ID on IQ scores measured at age 8 and 15 (presented in Table 7). Those with ID had IQ scores on average 40 points lower than those without ID at age 8 and 29 points on average lower at age 15. For those with probable ID the IQ scores were on average 30 points lower at age 8 and on average 21 points lower at age 15. It should be noted that, as IQ is included in the derivation of the multi-sourced variables, it is not surprising that IQ scores are lower among those indicated as having ID. Similarly, as two sources of information were required, ID/probable ID was not always indicated if IQ was less than 70/85. 

**Table 7.** Validation of derived ID variables against IQ measured at age 8 and 15.

	ID status	N	Mean IQ	SD	Range	Difference (95% CI)	p-value of difference ^a^
**IQ age 8**							
	No ID	7,04	104.49	16.12	53–151		
	ID	64	64.84	10.54	45–100	-39.65 (-35.69, -43.61)	<0.001
	No probable ID	6,755	105.62	15.43	53–151		
	Probable ID	349	75.33	8.21	45–100	-30.29 (-28.66, -31.92)	<0.001
**IQ age 15**							
	No ID	5,065	94.68	12.78	55–136		
	ID	50	66.14	9.82	55–92	-28.54 (-24.99, -32.09)	<0.001
	No probable ID	4,799	95.72	12.23	55–136		
	Probable ID	316	74.41	7.93	55–99	-21.31 (-19.94, -22.68)	<0.001

^a^ – two-sided p-value produced using t-test

### Known cause of ID flag

A comparison of those with a known cause of ID to those without a known cause of ID in terms of sources indicating ID is presented in Table 8. The table shows that 13 individuals identified as having ID had a known cause of ID. Those with a known cause of ID were more likely to be identified as having ID using free text information, SEN statements and GP records than those without a known cause of ID.

**Table 8.** Counts of those with/without ID who had a known cause of ID.

	ID (N=158)		No ID (N=13,736)	
	Known cause of ID, N(%)	No known cause of ID, N(%)	Known cause of ID, N(%)	No known cause of ID, N(%)
Total ^a^	13 (8.23)	145 (91.77)	18 (0.13)	13,718 (99.87)
Total with ID indicated by source ^b^				
IQ < 70 at age 8	≤5	47 (78.33)	≤5	74 (1.05)
IQ < 85 at age 8	≤5	57 (95.00)	≤5	795 (11.30)
IQ < 70 at age 15	≤5	33 (70.21)	≤5	105 (2.08)
IQ < 85 at age 15	≤5	43 (91.49)	≤5	1,160 (22.92)
Free text	7 (58.33)	44 (33.85)	≤5	39 (0.32)
SEN statement	12 (92.31)	93 (78.15)	≤5	163 (1.60)
GP Read code	9 (69.23)	73 (57.03)	≤5	71 (0.58)
HES ICD9/10 dx	≤5	≤5	≤5	≤5
MHSDS code	≤5	≤5	≤5	≤5
IDI project ICD-10 dx	≤5	74 (52.86)	≤5	31 (0.24)

Counts ≤5 may also include the value 0 ^a^ – Denominator for percentage equal to the number with ID/without ID ^b^ – Denominator for percentage equal to the number indicated by the Total row for the column who also had data available for the source of ID

Eighteen individuals who were not identified as having ID had a known cause of ID. This is possible as those with a genetic, metabolic, or chromosomal abnormality, used to identify known causes of ID, may not in fact develop an ID, or alternatively may not be investigated for ID as a result of their known abnormality. 

### Patterns of data availability and consenter status for linked health records

Consenter status for linked health records (GP records, HES data and MHSDS data) may also influence the ability to identify cases of ID. Table 9 presents the number of variables available to identify ID, the number with available IQ data and the average IQ scores at age 8 and 15, across categories of consent status. The non-explicit consenter group (those with section 251 approval) had on average one fewer available source of information (excluding linked health data sources) than the explicit consenters and were less likely to have available IQ measures at age 8 or 15 than those in the explicit consent or explicit non-consent groups. The non-explicit consenter group also had lower average IQ scores at age 8 and 15 than the explicit consenters. This may suggest that the non-explicit consenter group contains more severe cases of ID than the explicit consenters. It is important to note that the non-explicit consenter group is likely to include people who are unable to participate in ALSPAC, those who are unable to provide explicit consent as they lack capacity for this, or attend clinics to measure IQ, because of an ID.

**Table 9.** Descriptive statistics across categories of consent.

Statistic	Explicit consenter	Non-explicit consenter	Explicit non-consenter	No data linkage available
N	5063	7358	359	1590
N (%) with ID	38 (0.75)	103 (1.40)	≤5	15 (0.94)
N (%) with probable ID	175 (3.46)	227 (3.09)	20 (5.57)	27 (1.70)
Mean (SD) number of ID variables available ^a^	4.12 (1.10)	3.18 (1.04)	3.27 (0.99)	1.93 (0.84)
Median (IQR) number of ID variables available ^a^	4 (4-5)	3 (3-4)	3 (3-4)	2 (1-2)
N (%) with available IQ score at age 8	3838 (75.80)	2703 (36.74)	252 (70.19)	320 (20.13)
Mean (SD) IQ score at age 8 ^b^	107.64 (16.03)	99.94 (16.00)	103.13 (17.43)	97.81 (15.78)
N (%) with available IQ score at age 15	3475 (68.64)	1369 (18.61)	174 (48.47)	98 (6.16)
Mean (SD) IQ score at age 15 ^c^	96.16 (12.91)	90.56 (12.54)	93.09 (12.44)	87.87 (12.68)

^a^ Sources of data included were IQ at age 8 and 15, free text data, SEN statement and diagnosis in the IDI project (i.e. excluded linked health data) in order to be able to compare across consenter status. ^b^ Mean difference in IQ at age 8 between explicit consenter and non-explicit consenter sample = 7.69 points, two sided p-value for t-test = <0.001 ^c^ Mean difference in IQ at age 15 between explicit consenter and non-explicit consenter sample = 5.60 points, two sided p-value for t-test = <0.001

The impact of missing study data can be partially mitigated by the use of linked routine health and education records where available: however, each linkage source is also impacted by incomplete coverage. This means there are some ALSPAC participants for whom there exists insufficient evidence to assess ID status and that it is reasonable to suggest that disproportionate numbers of individuals with ID may fall into this group. Whilst this has not impacted the ascertainment of case status in those with information, it does mean that this case status should not be used to determine prevalence estimates and users of the data should note that some cases, possibly those with most pronounced ID, are missing from the data even where linked records are available.

## Ethics policies

Ethical approval for the study was obtained from the ALSPAC Law and Ethics committee and for the ALSPAC record linkage programme, from a local research ethics committees (NHS Haydock REC: 10/H1010/70). A comprehensive list of research ethics committee approval references is available to download.

## Consent

Written informed consent was obtained from the main caregiver of participating children after receiving a full explanation of the study. Children were invited to give assent where appropriate. Study members have the right to withdraw their consent for elements of the study or from the study entirely at any time. Full details of the ALSPAC consent procedures are available on the study website. Access to the linked health records of those who had not explicitly consented to data linkage was authorised via use of Section 251 of the National Health Service Act 2006 ^18^ for GP records but not HES or MHSDS data (CAG reference: 20.CAG/0056; IRAS project ID: 268410).

## Data Availability

ALSPAC data access is through a system of managed open access. The steps below highlight how to apply for access to ALSPAC data, including access to the data and R scripts described in this data note. Please read the ALSPAC access policy (PDF, 627kB) which describes the process of accessing the data and samples in detail, and outlines the costs associated with doing so. You may also find it useful to browse our fully searchable research proposals database, which lists all research projects that have been approved since April 2011. Please submit your research proposal for consideration by the ALSPAC Executive Committee. You will receive a response within 10 working days to advise you whether your proposal has been approved. If you have any questions about accessing data, please email alspac-data@bristol.ac.uk. The ALSPAC data management plan describes in detail the policy regarding data sharing, which is through a system of managed open access.
