# Supplementary material for: Intellectual disability in the children of the Avon Longitudinal Study of Parents and Children (ALSPAC)
Source: Wellcome Open Res. 2023 Jan 11;7:172. Originally published 2022 Jun 17. [Version 2] doi: 10.12688/wellcomeopenres.17803.2 (PMC10276197; doi:10.12688/wellcomeopenres.17803.2)
Supplement: Peer review history

# Peer review: Reviewer response for version 2

**DOI:** 10.21956/wellcomeopenres.20891.r59430

**Published:** 2023-06-16

**Authors:** Freya Tyrer

**Affiliations:** 1 Department of Health Sciences (Biostatistics Research Group), University of Leicester, Leicester, England, UK

Thank you for the opportunity to review this paper which I found very interesting and well-written. I agree that the use of multiple sources to identify ID is useful owing to the ambiguities in the definitions of ID.

 I have minor comments, as below:

- It is not surprising to me that ID is poorly identified in English HES data using F7x codes owing to coders' and clinician's preferences for the F81.9 (developmental disorders of scholastic skills) for ID - a recent publication has also highlighted this (see https://pubmed.ncbi.nlm.nih.gov/36940198/ ^1^). I can see why the authors haven't used this as it could potentially refer to people without ID, but I think there does need to be some recognition of this preference in the article - perhaps in the limitations section. There seems to be some reluctance to change these codes because hospitals receive an uplift for any interventions in patients with ID. I think the inability to identify ID based on HES data alone would be a useful recommendation arising from this work.
- The only other comments I had relates to Figure 1. The figure shows 93 people being identified from free text fields, but the narrative refers to 94 people. Similarly, 1289 people were excluded but the numbers of people excluded adds up to 1323. This might be because people fit into more than 1 category but it stands out (to me anyway). I'd recommend looking at the entire figure to make sure that everything is consistent and makes sense and that overlaps are specified, where they occur.

Are sufficient details of methods and materials provided to allow replication by others?

Yes

Is the rationale for creating the dataset(s) clearly described?

Yes

Are the datasets clearly presented in a useable and accessible format?

Yes

Are the protocols appropriate and is the work technically sound?

Yes

Reviewer Expertise:

Intellectual disability, biostatistics, big data

I confirm that I have read this submission and believe that I have an appropriate level of expertise to confirm that it is of an acceptable scientific standard.

## References

[1] : Recording of intellectual disability in general hospitals in England 2006-2019: Cohort study using linked datasets. *PLoS Med* .2023;20(3) : 10.1371/journal.pmed.1004117 e1004117 10.1371/journal.pmed.1004117 36940198PMC10069786

# Peer review: Reviewer response for version 2

**DOI:** 10.21956/wellcomeopenres.20891.r54047

**Published:** 2023-01-11

**Authors:** Valeria Frighi

**Affiliations:** 1 Department of Psychiatry, University of Oxford, Oxford, UK

Thanks for adding the figure requested to the abstract.

Are sufficient details of methods and materials provided to allow replication by others?

Yes

Is the rationale for creating the dataset(s) clearly described?

Yes

Are the datasets clearly presented in a useable and accessible format?

Yes

Are the protocols appropriate and is the work technically sound?

Yes

Reviewer Expertise:

Endocrine disorders, in particular in people with intellectual disabilities and people with severe mental illness

I confirm that I have read this submission and believe that I have an appropriate level of expertise to confirm that it is of an acceptable scientific standard.

# Peer review: Reviewer response for version 1

**DOI:** 10.21956/wellcomeopenres.19708.r53553

**Published:** 2023-01-04

**Authors:** Valeria Frighi

**Affiliations:** 1 Department of Psychiatry, University of Oxford, Oxford, UK

The authors, in creating and making available the datasets used to identify people with intellectual disabilities in the Avon Longitudinal Study of Parents and Children (ALSPAC), should be credited with two simultaneous achievements. The first is to show that the methods used to identify these subjects are scientifically sound, reliable and reproducible. The second is a real service to other researchers in the field of intellectual disabilities, who might use the same methods in their studies. I totally agree with the authors that whenever possible, a dual source of identification of people with intellectual disabilities should be used. This is particularly true when dealing with widely used but ambiguous terms such as “learning difficulties” or “special educational needs” or with people with IQ scores in the borderline region of 70-85.

 I only have one very minor suggestion in relation to the current publication, i.e. to add the number of individuals with a known cause of ID (n=31) also as a percentage (19.6%) of the total number of those with ID (n =158) to the summary. This information is currently found only within the text and in table 8 but it is important enough to be included in the summary.

 I then have two additional suggestions to the research group as such. One is to consider writing an opinion/review paper on the usefulness, or otherwise, to screen for genetic causes in all individuals with ID. I think this group would have the expertise for such a publication and that a paper of this type would be extremely useful.

 The second suggestion is to consider whether Tables 1 and 2 of the Supplementary Material of the paper by Frighi et al. ^1^ could be at all useful to improve detection of known causes of intellectual disabilities within ALSPAC.

Are sufficient details of methods and materials provided to allow replication by others?

Yes

Is the rationale for creating the dataset(s) clearly described?

Yes

Are the datasets clearly presented in a useable and accessible format?

Yes

Are the protocols appropriate and is the work technically sound?

Yes

Reviewer Expertise:

Endocrine disorders, in particular in people with intellectual disabilities and people with severe mental illness

I confirm that I have read this submission and believe that I have an appropriate level of expertise to confirm that it is of an acceptable scientific standard.

## References

[1] : Incidence of fractures in people with intellectual disabilities over the life course: a retrospective matched cohort study. *EClinicalMedicine* .2022;52: 10.1016/j.eclinm.2022.101656 101656 10.1016/j.eclinm.2022.101656 36313144PMC9596306
